# Effects of Student Training in Social Skills and Emotional Intelligence on the Behaviour and Coexistence of Adolescents in the 21st Century

**DOI:** 10.3390/ijerph18105498

**Published:** 2021-05-20

**Authors:** Sara Vila, Raquel Gilar-Corbí, Teresa Pozo-Rico

**Affiliations:** Department of Developmental Psychology and Didactics, University of Alicante, 03080 Alicante, Spain; vp.sara@hotmail.com (S.V.); raquel.gilar@ua.es (R.G.-C.)

**Keywords:** social skills, emotional intelligence, secondary school, educational training

## Abstract

In recent decades, efforts have been made to achieve a positive coexistence among adolescents in secondary schools and create a healthy environment to prepare them to face the present-day challenges. Therefore, this study highlights the educational purpose of improving emotional management and social skills as well as decreasing antisocial and criminal behaviour among secondary education students through an educational training programme. Accordingly, to verify the effectiveness of the project, a quasi-experimental design with a pre-test/post-test structure and a control group was adopted. To achieve this, a total of 141 Spanish secondary school students participated in this study and were randomly assigned to one of two experimental conditions. The first (experimental) group (*n* = 55) participated in the training programme; correspondingly, the second group (control) (*n* = 57) followed the usual mentoring activities planned for the entire educational centre. Of the total number of participants, 52.7% of the sample were men and 47.3% were women. The mean age of the participants was 13.01 years old (SD = 0.935). The results showed improvements in the environment with adequate training and the correct application of a programme involving emotional intelligence (EI) among secondary education students. Furthermore, a decrease in conflicts and enhanced relations between the members of the educational community was evidenced. Finally, the practical implications for improving coexistence in secondary schools are discussed.

## 1. Introduction

This study considers the analysis of and intervention for an increasingly common and widespread challenge in our current society: behavioural problems in school. The purpose of this article is twofold: on the one hand, to improve students’ emotional competencies and social skills and, on the other hand, to reduce criminal and antisocial behaviours. All of this aligns with the challenges of positive coexistence in 21st-century secondary education centres.

Thus, the research work focuses on secondary education students and the behavioural problems they exhibit within educational centres. The existing research highlights that there has been an increase in conflicts, school harassment, school violence, and disruptive behaviour in classrooms by students in the last decade [1,2,3]. It is also true that the health situation of the pandemic caused by the coronavirus disease 2019 (COVID-19) outbreak imposes serious restrictions on both the evolutionary development of the students as well as their motivation, social development, and academic progression [4,5,6]. Therefore, in these times of uncertainty, we require clear and front-line classroom proposals that will allow us to successfully overcome the situation and work among our students in these educational stages.

### 1.1. Theoretical Framework

#### 1.1.1. Intelligence: Conceptualisation, Theoretical Models, and Educational Implications

The conceptualisation of intelligence has evolved over time with a clear pedagogical impact in terms of educational implications and their consequent transfer to classroom work. This is especially the case in secondary education and is a key variable in the facilitation of other important aspects in academic performance such as motivation, satisfaction, and the assessment of intrinsic cognitive abilities. All of these factors have a great impact on current research that makes it possible to define successful pedagogical strategies [7,8,9].

At first, intelligence was considered to be a general and easily measurable construct from a psychometric point of view. This was the cradle of a compendium of tests with which students were academically evaluated, and important decisions were made about pertinent pedagogical orientation. These cases are still considered as indicators of the academic evolution of students, although they are no longer considered as the only premise to take into account when making decisions in the academic sphere [10,11,12,13,14,15,16].

In addition, these conceptions have a high impact on other variables such as the promotion of creativity in the classroom, respect for the individuality of students, the psychometric evaluation of students for academic orientation, and the commitment to cultivate effort as an important aspect for the progression of students in this key stage of their development [17,18,19].

In the same way, the cognitive theories in the conceptualisation of intelligence that rely on making an assessment of the different cognitive processes involved in intelligent and adaptive behaviour of great relevance in pedagogical approaches are especially noteworthy. Specifically, this applies to the secondary education stage, where it is necessary to detect the fundamental talents of the students and guide them towards the optimal management of their own intelligence for academic performance and personal enrichment. Moreover, this includes a clear commitment to respecting the emotional dimension of the students to aid in the optimisation of their learning [20,21,22].

Therefore, the scientific literature shows that including pedagogical designs in intelligence training related to emotions is necessary. This involves intrapersonal (self-knowledge and the successful management and regulation of one’s own inner world) skills and interpersonal (the necessary social competence to put oneself in the place of others and understand and manage their emotions and feelings) skills which are essential for full development at this educational stage [23,24,25].

In conclusion, the historical evolution of the conceptualisation of intelligence makes it possible for the pedagogical designs defined for the secondary education stage to have definitions of the meaning, purpose, and mission of each didactic proposal, considering that intelligence cannot be considered as a unitary construct. Rather, there are diverse talents, and they are combined in a unique way in each student. For these reasons, personalised attention, transversal work of diverse forms of intelligence from all areas of competence, giving prominence to the student in their own learning, educational methodology based on respect for diversity, and the use of a multiplicity of resources and formats favour the commitment to quality and excellence in the teaching used in educational settings. Accordingly, this study is absolutely compromised with these educational principles in mind.

#### 1.1.2. Social Skills, Emotional Intelligence (EI), and Learning Communities: Keys to the Wellbeing, Happiness, and Empowerment of Students

This section deals with the way in which secondary school students manage their emotions and social dimensions to achieve their wellbeing, personal progression, and positive socialisation. Therefore, the commitment of the teaching staff and the curricular design is fundamental to guarantee that the students have an appropriate didactic response to facilitate their growth as well as a feeling of belonging to a community along with empowerment in this crucial stage of their training.

The way in which adolescents perceive themselves depends to a great extent on the treatment they receive from other people; at this stage, popularity and acceptance amongst peers take on special relevance. Therefore, it is essential to develop the social skills necessary to be able to defend one’s own dignity and individual freedom, especially at this stage of development [26,27,28], but without attacking or denigrating the rights of others to achieve this goal.

Furthermore, in the process of perceiving one’s worth, the management of emotions carries a lot of weight. There are basic emotions found in all cultures such as joy, fear, shame, disgust, or anger. Moreover, common features are detected in emotional expressions at a cross-cultural level. In secondary school, it is especially important that students are able to identify their own and other’s emotions, have the vocabulary and the necessary skills to express them, and have enough skills to successfully handle them [29,30,31].

The fundamental role of emotions at a universal level is to preserve physical integrity. In this way, we find emotions that are pleasant and others that are negative or aversive—but in both cases the purpose is survival. For this reason, emotions as hard as fear allow us to preserve life in threatening situations. In the same way, anger urges the actions of defence and attack in situations in which an attack or a violation of fundamental rights is perceived. The way in which adolescents manage these universal emotions has a very important impact on their social skills, their relationship with their peers, the classroom climate, and their personal wellbeing [32,33,34].

In the same way, there is a widely confirmed relationship in the scientific literature between a lack of social skills and a low degree of emotional intelligence (EI) with academic social problems such as bullying and health, which are now as serious as depression, anxiety, or compulsive disorders [26,27,28].

It should be noted that, ultimately, the objective of secondary education is to be able to train individuals with the knowledge, skills, attitudes, and key and basic competencies needed for their life and personal development. In this manner and, in a prominent way, at the curricular level of this stage, the goal that students are able to fully enter adult life and achieve their happiness and wellbeing is pursued [35,36,37].

Therefore, it is important that the curricular designs at this stage provide secondary school students with the resources and opportunities necessary for their maturation and the provision of meaning to their academic and personal progression [38,39,40].

#### 1.1.3. Learning Communities: A Commitment to Favouring the Feeling of Belonging and Cultivating the Psychosocial Dimensions of the Students

The feeling of being appreciated, listened to, being part of a community (in this case, educational), and perceiving that personal needs are being attended to enable students to show better socialisation, use effective stress management, and express frustration along with high levels of wellbeing [41,42,43].

It should be emphasised that when the institute adopts a learning community dynamic, thus achieving a positive social climate, there should be no cases of bullying and the members of the same community should establish bonds of friendship, respect, and positive relationships. Subsequently, feelings of satisfaction in the academic environment by both students and teachers are high [44,45,46].

These benefits occur because in the context of a learning community, a multitude of transversal activities are carried out at an educational level that allow secondary school students to enhance their social skills and their emotional management in the face of conflicts [47,48,49].

It should be recognised that one of the most common difficulties in secondary education in our country is that each teacher is specialised in an area of knowledge, creating a risk that they will only work on the curricular contents of that area. However, the ideal in the framework of the mobilisation of the centre as a learning community is that teachers work as a team and make the students feel part of a social group made up of all the members of the educational institution—including families, students, administration staff, and teachers. In this way, relationships of mutual help, cooperation and ties of esteem, and a feeling of belonging are favoured [50,51,52].

Moreover, it is important that in the educational institutions of the 21st century an academic life dynamic based on the learning community model is achieved, focused on giving the students a leading role and committing to the acquisition of social skills, the emotional management of conflict situations, and the progression of the social and emotional dimension of individuals complemented by communication, contact, and a positive climate. This is also established through virtual learning environments, in addition to the typical face-to-face learning sessions in classrooms [53,54,55].

In this regard, we know that one of the basic needs of the individual is to feel that they are accepted and appreciated for who they are and to feel that they have an important role within their community, to establish bonds of loyalty, commitment, ethics, and cooperation. Moreover, they should obtain help in times of need, either on a personal level or to overcome the requirements of an academic subject [56,57,58]. These are the fundamental pillars for the achievement of academic performance and personal wellbeing in educational institutions within a dynamic of sustainable growth and commitment to full training.

It is very important that curricular designs in secondary schools include teaching strategies, whether based on the master class or mobilised through virtual learning environments. These designs should allow students to work in unison, have social skills to handle social situations in the classroom, and appropriately manage social experiences in which an adequate understanding, identification, expression, and regulation of one’s own and others’ emotions is required [59,60,61]. This way of betting on EI enables conflict prevention and the promotion of a positive classroom climate and social synergies among secondary school students.

Studies show that the educational centres that work best are those that offer resources, opportunities, and support for the wellbeing and the social and emotional growth of all their members. Moreover, these centres should be capable of combining discipline and the transmission of social norms with acceptance, appreciation of the individual, respect for diversity, and inclusion [62,63,64]. In this context, students have the opportunity to know their potential and their areas for improvement and the way in which they can articulate this to their peers to achieve optimised academic performance and wellbeing inside and outside of the classroom.

It should be emphasised that current scientific evidence shows that there is a multiplicity of knowledge that requires cooperative work to internalise and achieve a true impact on secondary school students. In fact, it is strongly indicated that learning is enhanced when it is done in a group and not in an isolated manner. This holds true as long as the teaching methodologies used are appropriate and allow students to share curricular content and mutually support each other in mastering the subject [65,66,67]. These principles are especially important in current educational institutions.

In addition, a positive relationship is confirmed between the educational dynamics based on the learning community and its eventual commitment to cultivating social skills. EI and the psychosocial dimension of students are key in the achievement of a resilient and strengthened attitude in the face of adverse situations or conflict [66,67,68,69,70]. In fact, the psychosocial adaptation of students provides for rapid improvement the sooner social skills and EI are worked on in educational curricula.

We can conclude that the possibility of working in learning communities within institutes encourages students to cultivate their social dimension, fostering engagement in an active social life, inclusion in the social environment, positive synergies between students, effective communication, regulation of emotions in contact with those of others, and personal wellbeing along with academic performance in educational settings.

In conclusion, the importance of working on emotions and secondary social skills is evidenced by its impact on academic performance, the feeling of belonging to the educational community, optimal socialisation, and the wellbeing of students at this stage [27,71,72]. Therefore, it is appropriate to include and mobilise social skills, EI, and learning communities in the pedagogical designs of educational institutions of the 21st century.

### 1.2. Teacher Training Programme

The 12-week training programme of the present study was designed to improve EI and social skills as well as to prevent criminal and/or antisocial behaviours among secondary school students. Table 1 describes the details of each lesson in the programme.

### 1.3. Objectives and Hypotheses

The general objective of this work is to analyse the effectiveness in the design and development of the intervention programme, “I live, therefore, I feel” for the improvement of (1) EI; (2) social skills; and (3) antisocial and criminal behaviour among secondary education students.

The general hypothesis is formulated as follows:

There are significant improvements in EI and in the behaviour of compulsory secondary education students who participate in the specifically designed EI programme.

In this regard, the specific objectives and hypotheses are the following:

Specific Objective 1: Evaluate the initial situation of the experimental and control groups in relation to socioemotional skills and antisocial and criminal behaviours.

**Hypothesis** **1.** *There are no significant differences in the pre-test moment between the experimental and control groups in the variables included in the study.*

Specific Objective 2: Analyse the changes produced in the study participants, depending on whether they belong to the experimental or the control group, in the following variables: (1) EI; (2) social skills; and (3) antisocial and criminal behaviour among secondary education students once the programme has been applied.

**Hypothesis** **2.** *The experimental group achieves a significant improvement compared to the control group in the variables included in the above-mentioned study.*

Finally, the research questions were formulated as follows:

(1) Is it possible to design and develop an effective intervention programme to improve EI and social skills among secondary school students?
(2) Is it possible to design and develop an effective intervention programme to prevent antisocial and criminal behaviours among secondary school students?
(3) What are the educational implications of the development of an effective intervention programme to improve the behaviour and coexistence of adolescents in the 21st century?

## 2. Materials and Methods

### 2.1. Participants

A total of 141 Spanish secondary school students participated in this study and were randomly assigned to one of two experimental conditions. The first (experimental) group (*n* = 55) participated in the training programme; correspondingly, the second group (control) (*n* = 57) followed the usual mentoring activities planned for the entire educational centre. Of the total number of participants, 52.7% of the sample were men and 47.3% were women. Regarding the ages of the participants in the study, as can be seen in Table 2, 33.9% were 12 years old; 39.3% were 13 years old; 19.6% were 14 years old; 6.3% were 15 years old; and only 0.9% were 16 years old. The mean age of the participants was 13.01 years old (SD = 0.935).

### 2.2. Instruments

The instruments used in the design and implementation of the programme are described below, in accordance with the objectives, hypotheses, and goals pursued in the investigation.

1. Emotional Quotient Inventory Youth Version (EQi:YV; 30-item) [73]: This instrument is a self-report designed to estimate the level of EI and the socio-affective profile of the subjects, specifically in children and adolescents between 12 and 18 years old. The questionnaire used consisted of 30 items distributed in the following scales: intrapersonal: refers to knowledge of oneself on an emotional level, resolution, independence, self-esteem, and self-realisation; interpersonal: focused mainly on empathy, social responsibility, and interpersonal relationships; stress management: covering areas such as stress tolerance or impulse control, among others; adaptability: refers to problem solving, flexibility, or reality testing. The response format was a gradual range from 1 to 4 that went as follows: 1 = “It is not true in my case”; 2 = “A little true in my case”; 3 = “It is true in my case”; and 4 = “Very true in my case”. The Spanish validation of EQi:YV (S) [74] had an adequate reliability of its scales and full scale, whose values ranged from 0.84 to 0.89, respectively.
2. Trait Meta-Mood Scale (TMMS-24) [75]: This is a questionnaire that has had a Spanish validation for more than a decade [76], and it was used in the present investigation. This scale was designed to assess how people reflect upon their moods and determine the extent to which people attend to and value their feelings (attention), feel clear rather than confused about their feelings (clarity), and use positive thinking to repair negative moods (repair). It was implemented first through 48 items and its adapted version (TMMS-24) through 21 items. The response range from 1 to 5 is as follows: 1 = “Not at all agree”; 2 = “Somewhat agree”; 3 = “Pretty much agree”; 4 = “Strongly agree”; and 5 = “Totally agree”. The predictive validity was similar to that of the original version and the reliability of the validation sample for each dimension went as follows: attention = 0.90; clarity = 0.90; and repair = 0.86.
3. Matson Evaluation of Social Skills in Youngsters (MESSY) [77]: This is an instrument designed to assess the degree of adequacy of social behaviour. In addition, it consists of two questionnaires, one to be completed by the students and the other by the teachers. This questionnaire measured appropriate skills and competencies and social behaviour problems. The MESSY is made up of 62 items organised into five factors: aggressiveness/antisocial Behaviour (AAB), social skills/assertiveness (SSA), conceit/haughtiness (CH), loneliness/social anxiety (LSA), and MESSY total scale. Regarding its response format, five gradual options were presented, ranging from 1 to 5 as follows: 1 = “It does not describe me at all (I am not like this at all)”; 2 = “It describes me a bit (I am a bit like this)”; 3 = “Describes something to me (I see myself as something like this)”; 4 = “It describes me a lot (I am like this a lot)”; and 5 = “It describes me a lot (I am like this)”. Recent studies show that MESSY has adequate psychometric properties, including internal consistency and convergent and divergent validity [78]—and always with the Spanish validation, as well [79].
4. Antisocial-Criminal Behaviour Questionnaire [80]: This questionnaire is made up of 2 subscales with 40 items in total that evaluate antisocial behaviour with 20 items and criminal behaviour with another 20 items. It is an instrument in which the subjects must read the sentences and report whether they have performed the behaviours they describe, using either a “yes” or “no” response format. An adequate scale structure and reliability analysis with a Cronbach’s alpha of 0.90 have been confirmed [81].

### 2.3. Procedure

The study was carried out throughout the 2018–2019 academic year. First, a meeting was requested with the management team and the educational centre’s guidance department to explain the work objective. In the meeting, it was reported that, in addition to designing and implementing a programme to improve social and emotional skills in experimental groups of students, various instruments would be administered as resources to statistically evaluate whether there are actually misadjusted behaviours and lack of emotions among said students, as well as evaluating the possible or no improvement of said behaviours after finishing the project. For this, a first draft of the programme was designed.

After the acceptance and positive reception of the proposal, the director created a document to authorise the development of the project in the educational centre.

Likewise, a second meeting was held with the team of teachers/tutors of the first and second years of compulsory secondary education and the guidance departments, where the new objective, development, and purpose of the programme were described. Once all those present supported the project, the guidance department, in consensus with the teachers, proposed the appropriate groups for the development of the programme, resulting in the choice of two groups from the first year and two from the second year of secondary education.

Next, an explanatory meeting was held with the teaching staff regarding the possible participants and another with the families, with the aim of obtaining authorisation for the participate of the students in the project as supervised by the centre. It should be noted that of the leading groups that made up the sample, there was only one abstention.

Subsequently, the selected questionnaires were computerised and prepared in paper format. For this research, the TMMS-24, EQi:YV(S), MESSY, and the Antisocial-Criminal Behaviour Questionnaire instruments were chosen. In coordination with the teachers of the different groups, the dates were established to conduct the first phase of the project, the pre-test, and an indicative calendar for the development of the programme.

The assessment instruments were administered during November and December (pre-test phase). After making contact with the students and becoming aware of the needs they presented, during January the design and preparation were completed in paper format of the EI programme, “I live, therefore, I feel”.

Before the beginning of the programme, prior to the completion of the questionnaires by the participants, the tutors of each of the courses informed the students that they were going to administer questionnaires prior to the start and at the end of the programme. They informed the students that this would be done with varied questions, among which those related to group relations and capacities in IE stood out. Students were also advised that maximum assistance was required during the project period in order to take part in the research. 

The EI project was implemented during February, March, and April in the academic tutorial hours of the experimental groups. This programme is made up of two notebooks, one for the teachers and another for each student. It was distributed in 10 chapters which included the following basic skills of EI: perception, assimilation, understanding, and emotional regulation through dynamic, relaxed, and playful activities. All of the chapters started with a theoretical description of EI and/or an ability of it. Next, the activities to be carried out were presented and ended with a relaxation time. At the end of each session, experiences and opinions about the work were shared with the participants.

Once the “I live, therefore, I feel” programme was completed, the subsequent phase was carried out throughout May with the administration of the aforementioned instruments.

As of June, once the data collection phase obtained during the pre-test and post-test phases of the experimental and control groups had been completed, the ordering and coding of the same continued. To achieve this, all the information collected was entered in a spreadsheet utilising IBM’s SPSS version 22.0 software programme in order to proceed with its decoding, analysis, comparison, and the preparation of the corresponding tables.

### 2.4. Experimental Design and Data Analysis

A descriptive and correlational design was used to analyse the relationships between the study variables. To verify the effectiveness of the created project, a quasi-experimental design with a pre-test/post-test design and control group was adopted. The analyses used were the general linear model (GLM) of repeated measures. The software programme used for the analysis and graphic representation of the data was IBM’s SPSS version 22.0 licenced by the University of Alicante (Spain). Finally, all of the procedures were approved by the University of Alicante Ethics Committee (UA-2015-07-06).

## 3. Results

First, we proceeded to check whether the experimental and control groups presented significant differences in the variables considered in our study. For this, a means comparison analysis was conducted for the independent samples. The results of the comparison of means are presented in Table 2. As can be seen, most of the variables do not show significant differences between the experimental group and the control except for the variables’ adaptability. The total score (involved in the EQi), attention, clarity, and regulation (involved in the TMMS) were calculated, in which the control group and the variables aggression/antisocial behaviour (involved in MESSY) and the antisocial variable (involved in the Antisocial-Criminal Behaviour Questionnaire) scored higher.

Second, regarding the factors involved in the EQi questionnaire, the Box’s M-test result indicates the homogeneity of the variance-covariance matrices for the intrapersonal factor (F = 0.137; gl = 2,272,194.612; and *p* = 0.938) and the interpersonal factor (F = 0.317; gl = 2,272,194.612; and *p* = 0.813); stress management (F = 0.338; df = 2,272,194.612; and *p* = 0.978); adaptability (F = 1.560; df = 2,272,194.612; and *p* = 0.197); and EQi total score (F = 1.397; df = 2,272,194.612; and *p* = 0.242). Regarding the factors involved in the TMMS, the Box’s M-test test result indicates the homogeneity of the variance-covariance matrices for the attention factor (F = 2.033; df = 2,272,194.612; and *p* = 0.107); emotions clarity (F = 1.516; df = 2,272,194.612; and *p* = 0.208); and repair (F = 0.487; df = 2,272,194.612; and *p* = 0.691). Regarding the factors involved in MESSY, the Box’s M-test result indicates the homogeneity of the variance-covariance matrices for the SSA factor (F = 1.100; df = 2,272,194.612; and *p* = 0.348); CH (F = 0.234; df = 2,272,194.612; and *p* = 0.873) and MESSY total scale (F = 1.265; df = 2,272,194.612; and *p* = 0.284). However, the result does not indicate such homogeneity for the following variables involved in MESSY: factor AAB (F = 6.532; df = 2,272,194.612; and *p* = 0.000) and LSA (F = 5.515; df = 2,272,194.612; and *p* = 0.001). In the same way, the result does not indicate such homogeneity for the following variables in MESSY: factor AAB (F = 6.532; df = 2,272,194.612; and *p* = 0.000) and LSA (F = 5.515; df = 2,272,194.612, and *p* = 0.001) and in all factors involved in the Antisocial-Criminal Behaviour Questionnaire: antisocial behaviour (F = 3.924; df = 2,272,194.612; and *p* = 0.008) and criminal behaviour (F = 27.630; df = 3,278,777.941; and *p* = 0.000). In any case, it should be remembered that a violation of this assumption has a minimum effect if the groups are approximately equal in size [82].

Finally, the values resulting from intra-individual and inter-subject effects are presented in Table 3. These values show that the effect of the interaction between the evaluation time (pre-test and post-test) and the implementation of the educational intervention is significant (*p* = <0.05) for the students involved in the experimental condition compared to the control group, showing an improvement for the following dimensions:

1. A significant improvement in the level of EI:
  1.1. Confirmed through the EQi:YV (S) for the following dimensions: interpersonal, adaptability, and for the total emotional intelligence scale.
  1.2. Confirmed through the TMMS for the three dimensions: attention, clarity, and repair.
2. A significant improvement in the level of social skills, evidenced through the MESSY for the dimensions of aggressiveness and antisocial behaviour, as well as for social skills and assertiveness and, along the same lines, for the full scale.
3. A significant improvement in both antisocial and criminal behaviour measured through the Antisocial-Criminal Behaviour Questionnaire.

The rest of the factors measured do not show significant differences between the groups.

**Figure 1:**
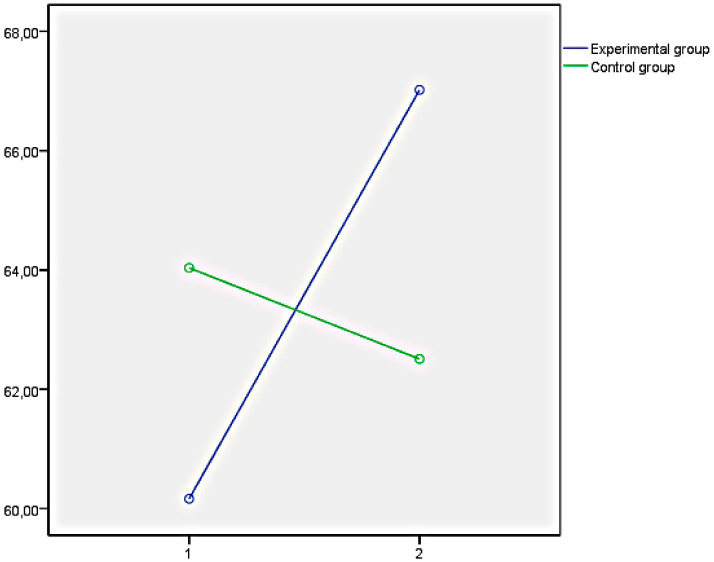
EQi total score as a representation of the significant differences obtained in a set of factors of EI measured with the Emotional Quotient Inventory Youth Version (EQi:YV).

**Figure 2:**
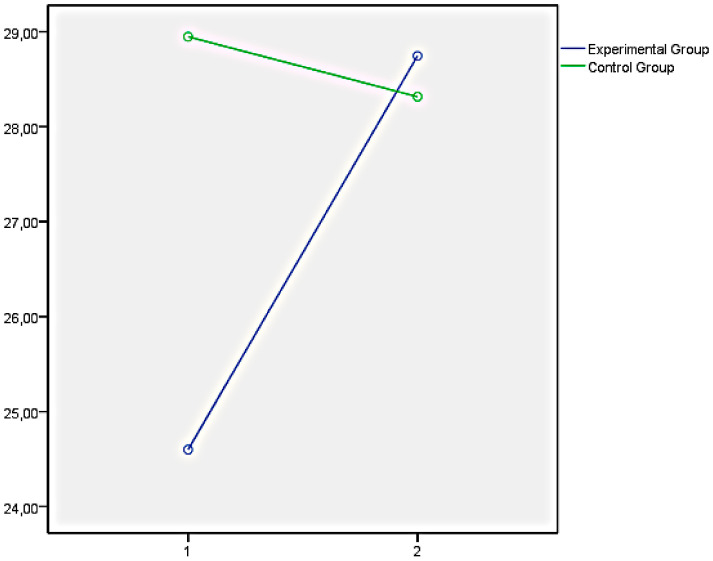
Attention after the educational intervention measured with Trait Meta-Mood Scale.

**Figure 3:**
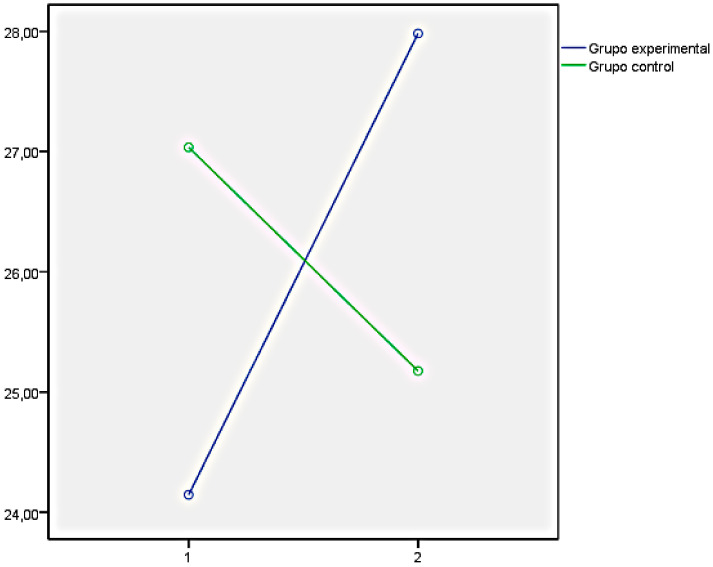
Clarity after the educational intervention measured with Trait Meta-Mood Scale.

**Figure 4:**
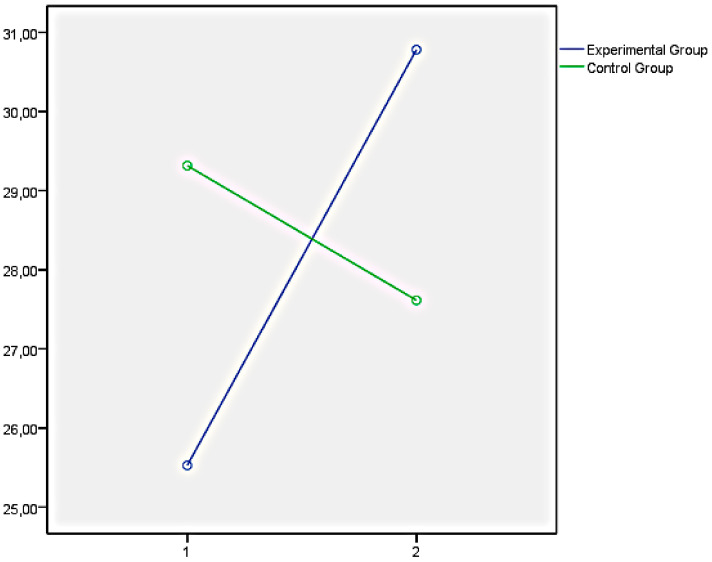
Repair after the educational intervention measured with Trait Meta-Mood Scale.

**Figure 5:**
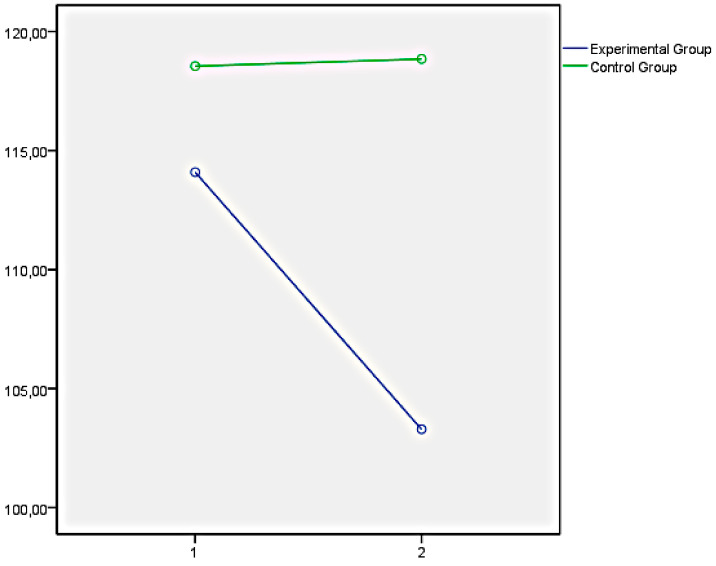
MESSY total score as a representation of the significant differences obtained in the set of factors of social skills measured with Matson Evaluation of Social Skills with Youngsters.

**Figure 6:**
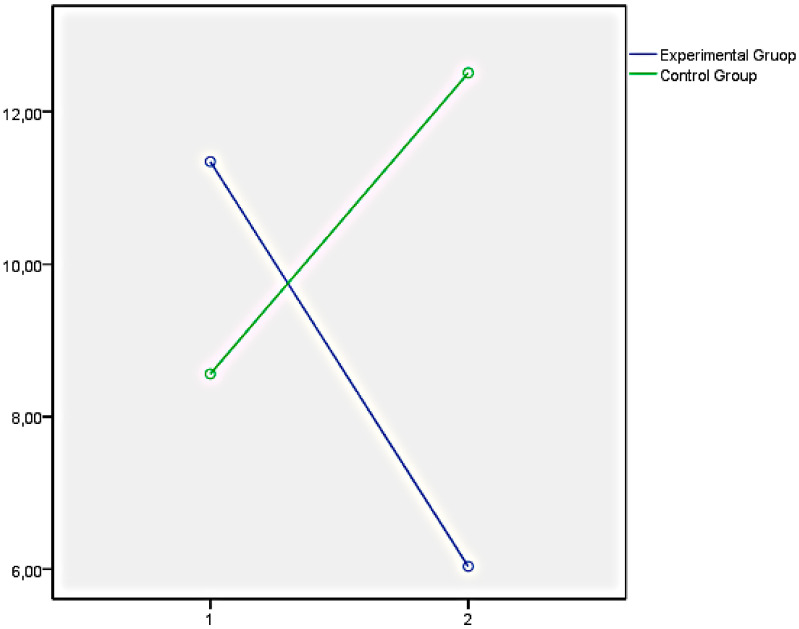
Antisocial behaviour after the educational intervention measured with the Antisocial-Criminal Behaviour Questionnaire.

**Figure 7:**
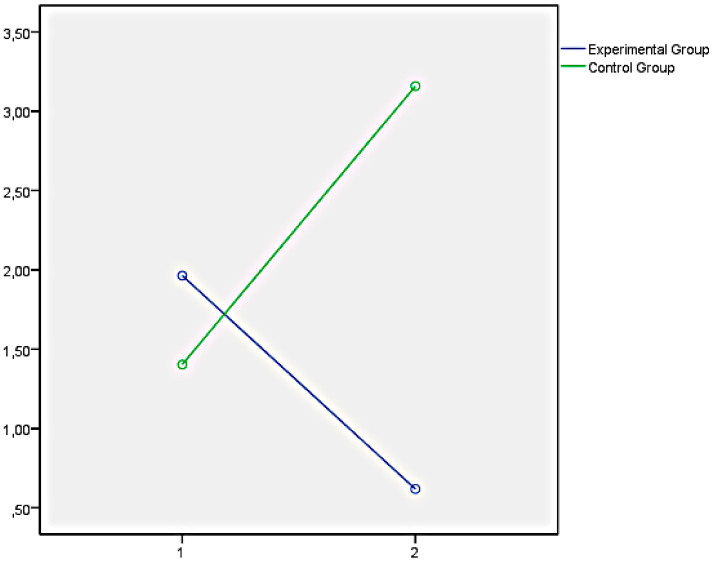
Criminal behaviour after the educational intervention measured with the Antisocial-Criminal Behaviour Questionnaire.

Finally, the graphs of the variables that showed significant differences between both groups have been presented below. In this regard, Figure 1 illustrates the EQi total score as a representation of the significant differences obtained in the set of factors of EI measures with the EQi:YV. Figure 2, Figure 3 and Figure 4 illustrate the EI factors after the educational intervention measures with the TMMS (attention, clarity, and repair, respectively). Next, Figure 5 illustrates the MESSY total score as a representation of the significant differences obtained in the set of factors of social skills measured with the MESSY. Lastly, Figure 6 and Figure 7 illustrate antisocial and criminal behaviour after the educational intervention measures with the Antisocial-Criminal Behaviour Questionnaire.

In all cases, the directions of the significant differences that were found presented a clear improvement within the experimental group after the training programme among the secondary education students.

## 4. Discussion

The measurement of each variable with the aforementioned instruments allows for a robust psychometric approach to evaluate the effectiveness of the programme in secondary school students. This is in line with the evidence provided in other programmes [77]; also, it allows us to ensure rigour in pedagogical interventions when working with adolescents, especially in those cases in which they present behavioural problems or disorders [78,83].

In fact, the multitude of scientific studies that have been undertaken on this subject in recent decades highlight the relevance of interventions of this type, as well as their impact in achieving pedagogical and academic objectives. Furthermore, they are related to the wellbeing and positive prospects of students [84,85,86].

According to the results obtained, and in response to the first and second research questions, it can be affirmed that is possible to design and develop an effective intervention programme to improve EI and social skills and to prevent antisocial and criminal behaviours among secondary school students. This study has shown that with adequate training and the correct application of a programme of emotional education and social skills, the results are satisfactory since improvements in the environment are appreciated. There is a decrease in conflict, students see their EI increase, and, consequently, academic results improve, which is in line with the evidence from other studies in the same field [87,88,89].

Furthermore, related to the third research question, the educational implications of the development of an effective intervention programme to improve the behaviour and coexistence of adolescents are relevant as the application of these programmes facilitates the improvement of relationships among members of the educational community and reduces cases of disruptive behaviour or problems in the relationships among peers. This is similar to the evidence provided by other studies [90,91,92].

In addition, the logical relationship between the development of social-emotional skills, such as those trained in this programme, and the high-value benefits to the dynamics of the school is evidenced [93,94,95].

However, our study presents limitations and indicates future research lines. The first of the clear limitations is that the programme was not effective for the intrapersonal and stress management scales. This may be due to the fact that the duration of the programme and its content are insufficient to improve EI at an intrapersonal level to adequately manage stress and to regulate mood. Future studies could increase the number of weeks of the programme and also include specific sessions with exact and operational tasks aimed at improving these three factors. In this way, it will be possible to evaluate whether students who are more successful at implementing the types of exercises developed or investing time in them achieve significant results at the level of those obtained in the other dimensions.

A second limitation is that the study sample could be larger to assess the results more accurately. Moreover, we worked with students from a single school who are already grouped by the classes of their academic year. Given this, there is a risk that this may condition the results in the face of a more psychometrically ideal situation where the programme can be implemented without previous groupings. In fact, it is possible that the idiosyncrasy of a class made up of students who already previously know one other and have shared experiences may determine the obtained results. In future studies, an increase in the sample size is planned by covering a greater number of secondary schools, as well as through grouping the students in a random way. Hopefully, through all these actions, greater rigour at the statistical level will be realised.

The third limitation is that the study could have considered other variables that may shed more light on the effectiveness of the programmes for other variables, such as present academic performance, health and personal wellbeing, and empowerment among secondary school students. This would be more in line with other current studies if a greater number of variables were to be contemplated within the experimental designs. The inclusion of these variables in future studies is a chance to determine the impact and predictive value of the programme on key aspects of the prospects of the students.

Finally, the methodology used is entirely classical and academic, which may represent a limitation of the programme. In the past few decades, training programmes have been created that take advantage of information and communications technology (ICT) and the facilities of e-learning education alternatives to improve their proposals. This includes innovative methodological approaches such as the present use of transversal educational platforms to reinforce and enrich the work of the sessions in a face-to-face format, the inclusion of augmented reality in the proposals, or virtual teaching and learning environments.

## 5. Conclusions

Across the present study, we have verified our hypothesis: effectively, there were significant improvements in EI and social skills and a decrease in antisocial and criminal behaviours among the compulsory secondary education students who participated in the specifically designed training, this being a priority direction of contemporary youth education.

The top practical application of this research is that, across an effective EI training programme among students, it is possible to achieve a pluralistic and democratic secondary school centre where the rights of all members of the educational community are inalienable. In addition, this educational programme allowed the promotion of axial values for harmonious coexistence.

On the other hand, there are some requirements that must be met to achieve improvements in EI, social skills, and criminal behaviour among high school students within this type of educational intervention. The acceptance and positive reception of the proposal by the entire educational community, teachers involved, families, students, and other educational stakeholders (academic guidance teams and academic members of the community, in general) is key. That is why the first step of this research was based on the creation of a document to authorise the development of the project in the secondary school centre.

Another important requirement is to include in the programme design a clarification of the goals, content, schedule, methodology, and evaluation to guide teaching practice across the implementation of the programme. Even more important, the involved teachers must acquire teaching skills to motivate students to learn EI and social skills, show a sincere interest in students, and know how to adapt to changes. In other words, the teaching commitment to the programme is key to achieving the desired objectives.

In conclusion, we can affirm that the proposed programme improved the emotional competencies and social skills of the students. In addition, it reduced criminal and antisocial behaviours, proving itself as effective and as a commitment to the improvement of and excellence in education in the 21st century.

## Figures and Tables

**Table 1:** Secondary school students training programme called “I live, therefore, I feel”.

	Lesson	Target	Tasks
1	Pre-test Phase	Measurement instrument compliance before the training.	Evaluate EI, social skills, and criminal and/or antisocial behaviours
2	“Discovering emotions”	− Know and identify different emotions. − Learn to control breathing.	Presentation Emotions brainstorming Dictionary of emotions
3	“Identifying emotions”	− Recognise and consolidate various emotions and emotional states in themselves and in others. − Learn to control and relax the body.	How do I feel? How do others feel? I feel my body
4	“I feel”	− Discover and identify emotions that are manifested in various situations. − Learn to relax after situations that generate anxiety.	What I feel when... My body talks! My worlds
5	“I have fun with emotions”	− Recognise one’s own emotions and those of others that arise in various situations. − Communicate properly. − Learn and control to focus attention.	I connect with my 90-year-old self Unknown partner Attention, let’s paint!
6	“I understand myself”	− Understand different emotions and emotional states. − Identify and know secondary emotions. − Practise guided relaxation and visualisation.	I self-portrait! Tell me a story I travel without moving
7	“We understand each other”	− Reasoning using emotions appropriately. − Analyse objectively. − Know how to put oneself in the place of the other. − Discover postures that facilitate relaxation.	We’re going to the theatre “Poseur” activity
8	“And why am I behaving this way?”	− Know how to control positive and negative emotions. − Be able to focus attention on various parts of the body to relax them.	We analyse ourselves Shall we dance? Autogenic training (hot/cold, light/heavy, etc.)
9	“We take action!”	− Control and manage one’s own positive and negative emotions and those of others. − Use different types of music to achieve relaxation.	The Big Brainstorming Positive emotions May the music be with us
10	“We live with emotions”	− Be able to make creations influenced by emotions.	Fantastic binomial The musical carousel
11	“Reviewing what has been learned”	− Promote and strengthen self-esteem. − Discover how postural control favours relaxation.	My WhatsApp profile Do you dare to dream? Mitchell’s method
12	Post-test Phase	Measurement instrument compliance after the training.	Evaluate EI, social skills, and criminal and/or antisocial behaviours.

**Table 2:** Student’s *t*-test results for difference in mean scores (before training).

Variables	t	gl	Sig.	Difference	SD
Intrapersonal intelligence	−1.30	110.00	0.20	−1.16	0.89
Interpersonal intelligence	−1.51	110.00	0.13	−1.03	0.68
Stress management	−0.73	110.00	0.47	−0.43	0.59
Adaptation	−2.16	110.00	0.03	−1.26	0.58
EQi total	−2.22	110.00	0.03	−3.87	1.75
Attention	−3.16	110.00	0.00	−4.35	1.38
Clarity	−2.55	110.00	0.01	−2.89	1.13
Repair	−2.77	110.00	0.01	−3.79	1.37
Aggressiveness/antisocial behaviour (AAB)	2.48	97.31	0.02	7.27	2.93
Social skills/assertiveness (SSA)	−0.96	110.00	0.34	−2.41	2.51
Conceit/haughtiness (CH)	1.24	110.00	0.22	0.81	0.65
Loneliness/social anxiety (LSA)	−0.68	110.00	0.50	−0.37	0.55
MESSY total scale	−1.14	110.00	0.26	−4.45	3.90
Antisocial behaviour	3.06	106.16	0.00	2.78	0.91
Criminal behaviour	1.24	110.00	0.22	0.56	0.45

**Table 3:** Results of intra-subject/inter-subject univariate analysis of variance (ANOVA).

Area Examined	Source	Type III	df	F	Sig.	Partial η^2^	Ob. Power
Emotional Intelligence Scores (Emotional Quotient Inventory)							
Intrapersonal intelligence	Intra	32.01	1.00	2.69	0.10	0.02	0.37
	Intra*Inter	39.86	1.00	3.36	0.07	0.03	0.44
	Error intra	1306.88	110.00				
	Inter	40,821.11	1.00	1490.30	0.00	0.93	1.00
	Condition	5.46	1.00	0.20	0.66	0.00	0.07
	Error inter	3013.03	110.00				
Interpersonal intelligence	Intra	60.63	1.00	13.09	0.00	0.11	0.95
	Intra*Inter	176.88	1.00	38.18	0.00	0.26	1.00
	Error intra	509.62	110.00				
	Inter	83,152.87	1.00	5154.85	0.00	0.98	1.00
	Condition	31.55	1.00	1.96	0.16	0.02	0.28
	Error inter	1774.41	110.00				
Stress management	Intra	1.72	1.00	0.47	0.49	0.00	0.10
	Intra*Inter	5.58	1.00	1.52	0.22	0.01	0.23
	Error intra	402.31	110.00				
	Inter	50,827.94	1.00	3982.89	0.00	0.97	1.00
	Condition	0.72	1.00	0.06	0.81	0.00	0.06
	Error inter	1403.77	110.00				
Adaptation	Intra	26.81	1.00	7.81	0.01	0.07	0.79
	Intra*Inter	87.95	1.00	25.63	0.00	0.19	1.00
	Error intra	377.44	110.00				
	Inter	54,451.25	1.00	3801.67	0.00	0.97	1.00
	Condition	0.00	1.00	0.00	0.99	0.00	0.05
	Error inter	1575.53	110.00				
EQi Total Score	Intra	397.33	1.00	12.39	0.00	0.10	0.94
	Intra*Inter	983.03	1.00	30.66	0.00	0.22	1.00
	Error intra	3526.52	110.00				
	Inter	900,986.68	1.00	7670.84	0.00	0.99	1.00
	Condition	5.70	1.00	0.05	0.83	0.00	0.06
	Error inter	12,920.16	110.00				
Emotional Intelligence Scores (Trait Meta-Mood Scale)							
Attention	Intra	172.81	1.00	6.01	0.02	0.05	0.68
	Intra*Inter	319.38	1.00	11.11	0.00	0.09	0.91
	Error intra	3162.05	110.00				
	Inter	171,225.09	1.00	2565.08	0.00	0.96	1.00
	Condition	214.81	1.00	3.22	0.08	0.03	0.43
	Error inter	7342.74	110.00				
Clarity	Intra	54.69	1.00	3.06	0.08	0.03	0.41
	Intra*Inter	454.08	1.00	25.38	0.00	0.19	1.00
	Error intra	1968.20	110.00				
	Inter	152,360.67	1.00	2660.35	0.00	0.96	1.00
	Condition	0.10	1.00	0.00	0.97	0.00	0.05
	Error inter	6299.79	110.00				
Repair	Intra	176.66	1.00	7.16	0.01	0.06	0.76
	Intra*Inter	677.25	1.00	27.44	0.00	0.20	1.00
	Error intra	2715.18	110.00				
	Inter	179,465.48	1.00	2603.10	0.00	0.96	1.00
	Condition	5.39	1.00	0.08	0.78	0.00	0.06
	Error inter	7583.73	110.00				
Social Skills (Matson Evaluation of Social Skills with Youngsters (MESSY)							
AAB	Intra	25.95	1.00	4.09	0.05	0.04	0.52
	Intra*Inter	1199.17	1.00	188.90	0.00	0.63	1.00
	Error intra	698.29	110.00				
	Inter	20,693.18	1.00	613.49	0.00	0.85	1.00
	Condition	190.39	1.00	5.64	0.02	0.05	0.65
	Error inter	3710.35	110.00				
SSA	Intra	1257.21	1.00	24.52	0.00	0.18	1.00
	Intra*Inter	1935.07	1.00	37.75	0.00	0.26	1.00
	Error intra	5638.93	110.00				
	Inter	2,722,732.10	1.00	11053.37	0.00	0.99	1.00
	Condition	672.10	1.00	2.73	0.10	0.02	0.37
	Error inter	27,095.86	110.00				
CH	Intra	0.26	1.00	0.06	0.80	0.00	0.06
	Intra*Inter	2.13	1.00	0.53	0.47	0.00	0.11
	Error intra	446.58	110.00				
	Inter	30,122.71	1.00	1590.86	0.00	0.94	1.00
	Condition	56.38	1.00	2.98	0.09	0.03	0.40
	Error inter	2082.84	110.00				
LSA	Intra	11.04	1.00	2.58	0.11	0.02	0.36
	Intra*Inter	1.02	1.00	0.24	0.63	0.00	0.08
	Error intra	469.82	110.00				
	Inter	55,785.35	1.00	5171.87	0.00	0.98	1.00
	Condition	3.22	1.00	0.30	0.59	0.00	0.08
	Error inter	1186.49	110.00				
MESSY Total Score	Intra	1543.52	1.00	24.50	0.00	0.18	1.00
	Intra*Inter	1723.84	1.00	27.36	0.00	0.20	1.00
	Error intra	6930.36	110.00				
	Inter	2,894,469.04	1.00	3603.54	0.00	0.97	1.00
	Condition	5600.54	1.00	6.97	0.01	0.06	0.74
	Error inter	88,355.25	110.00				
Antisocial-Criminal Behaviour Scores (Antisocial-Criminal Behaviour Questionnaire)							
Antisocial Behaviour	Intra	25.95	1.00	4.09	0.05	0.04	0.52
	Intra*Inter	1199.17	1.00	188.90	0.00	0.63	1.00
	Error intra	698.29	110.00				
	Inter	20,693.18	1.00	613.49	0.00	0.85	1.00
	Condition	190.39	1.00	5.64	0.02	0.05	0.65
	Error inter	3710.35	110.00				
Criminal Behaviour	Intra	2.34	1.00	1.47	0.23	0.01	0.22
	Intra*Inter	134.48	1.00	84.29	0.00	0.43	1.00
	Error intra	175.50	110.00				
	Inter	714.13	1.00	79.13	0.00	0.42	1.00
	Condition	54.85	1.00	6.08	0.02	0.05	0.69
	Error inter	992.71	110.00				

## Data Availability

The data presented in this study are available on request from the corresponding author.
